# Bibliographical

**Published:** 1894-04

**Authors:** 


### EDITORIAL.

### Bibliographical.

THE SANITARIAN. The Sanitarian is a monthly magazine devoted to the promotion of the art and science of sanitation, mental and physical in all their relations. By an investigation, presentation and discussion of all subjects in this large domain, as related to personal and household hygiene, domicile, soil and climate, food and drink, mental and physical culture, habit and exercise, occupation, vital statistics, sanitary organization and laws, in short, everything promotive of or in conflict with health.

Its purpose is to render sanitation a popular theme of study and universally practical. We regard the Sanitarian as one of the best, if not the very best publication of the kind in America, and it has certainly been the means of accomplishing great good. It is a publication in which all who have any interest in public health and welfare should have within reach, and should aid in its distribution amongst the people.

It is published in New York. Dr. A. N. Bell, Editor, Brooklyn, N. Y. The American News Co. is the agent. Subscription price, $4 00 per year.

---

TRAVER'S DENTAL EXAMINATION REGISTER FOR THE PROPER RECORDING OF EXAMINATIONS OF THE MOUTH, AND OF OPERATIONS UNDER WAY AND COMPLETED.

This is a very complete work for this purpose and enables any one to make complete record of the condition of any case that may be in hand. Every peculiarity, whether of decay, disease or otherwise, may be most perfectly recorded in this work with the least possible expenditure of time. All such records are invaluable for future reference, and especially if they are faithfully kept and brought up in the future as examination may be made of the several cases from time to time. Keeping such record has been altogether too much neglected by the profession, and those very interesting and instructive cases pass from sight and knowledge, and the lessons that ought to be learned in this way are lost. We know of no record book so complete in all respects as this. We feel confident that those who will test its utility will find in it about all they could desire.

It can be procured of the Dental Supply Co., Racine, Wis., and we presume of any Dental dealer.

